# Definition and analysis of gray matter atrophy subtypes in mild cognitive impairment based on data-driven methods

**DOI:** 10.3389/fnagi.2024.1328301

**Published:** 2024-06-04

**Authors:** Baiwen Zhang, Meng Xu, Qing Wu, Sicheng Ye, Ying Zhang, Zufei Li, for the Alzheimer’s Disease Neuroimaging Initiative

**Affiliations:** ^1^Institute of Information and Artificial Intelligence Technology, Beijing Academy of Science and Technology, Beijing, China; ^2^Faculty of Information Technology, Beijing University of Technology, Beijing, China; ^3^International College, Beijing University of Posts and Telecommunications, Beijing, China; ^4^Department of Otorhinolaryngology, Head and Neck Surgery, Beijing Chaoyang Hospital, Capital Medical University, Beijing, China

**Keywords:** mild cognitive impairment, subtype, mixture of experts, magnetic resonance imaging, longitudinal analysis

## Abstract

**Introduction:**

Mild cognitive impairment (MCI) is an important stage in Alzheimer’s disease (AD) research, focusing on early pathogenic factors and mechanisms. Examining MCI patient subtypes and identifying their cognitive and neuropathological patterns as the disease progresses can enhance our understanding of the heterogeneous disease progression in the early stages of AD. However, few studies have thoroughly analyzed the subtypes of MCI, such as the cortical atrophy, and disease development characteristics of each subtype.

**Methods:**

In this study, 396 individuals with MCI, 228 cognitive normal (CN) participants, and 192 AD patients were selected from ADNI database, and a semi-supervised mixture expert algorithm (MOE) with multiple classification boundaries was constructed to define AD subtypes. Moreover, the subtypes of MCI were obtained by using the multivariate linear boundary mapping of support vector machine (SVM). Then, the gray matter atrophy regions and severity of each MCI subtype were analyzed and the features of each subtype in demography, pathology, cognition, and disease progression were explored combining the longitudinal data collected for 2 years and analyzed important factors that cause conversion of MCI were analyzed.

**Results:**

Three MCI subtypes were defined by MOE algorithm, and the three subtypes exhibited their own features in cortical atrophy. Nearly one-third of patients diagnosed with MCI have almost no significant difference in cerebral cortex from the normal aging population, and their conversion rate to AD are the lowest. The subtype characterized by severe atrophy in temporal lobe and frontal lobe have a faster decline rate in many cognitive manifestations than the subtype featured with diffuse atrophy in the whole cortex. APOE ε4 is an important factor that cause the conversion of MCI to AD.

**Conclusion:**

It was proved through the data-driven method that MCI collected by ADNI baseline presented different subtype features. The characteristics and disease development trajectories among subtypes can help to improve the prediction of clinical progress in the future and also provide necessary clues to solve the classification accuracy of MCI.

## 1 Introduction

Mild cognitive impairment (MCI) is a stage between Alzheimer’s disease (AD) and cognitive normal (CN; Knopman et al., 2021). It is widely accepted that most late-onset AD patients originate from the MCI process. Previous MCI studies focused on gray matter atrophy, distinguishing MCI from AD or CN, and predicting MCI conversion. However, the current classification or prediction of conversion for MCI leaves much to be desired (van Oostveen and de Lange, 2021). This can be attributed primarily to two factors: First, when compared to AD, the disparity in cortical atrophy between MCI and CN individuals is relatively minor, leading to decreased accuracy in classification. Second, the variability in brain structures and disease progression among MCI patients complicates the classification and prediction of conversion, as not all MCI cases are homogeneous (Dubois and Albert, 2004; Bondi et al., 2014). Exploring the distinctions among MCI subtypes can deepen our insight into the cortico-cerebral, pathological, and neuropsychological aspects of MCI. This knowledge can offer crucial references for improving the accuracy of predicting MCI conversion and classification.

Studies on AD subtypes are theoretically grounded in the autopsy findings of Murray et al., which identified three distinct AD subtypes: typical AD, hippocampal-sparing AD, and limbic-predominant AD (Murray et al., 2011). Recent studies have supported the definition of on AD subtypes through cortical atrophy aligning with three identified subtypes (Zhang et al., 2021). However, the use of autopsy results to understand the majority of MCI patients is limited. This limitation arises because of factor such as their extended survival, the intricate progression of their condition, and the nuanced differences between CN individuals. Moreover, the dependence on cognitive scales for subtype discrimination could be influenced by patients’ educational and cultural backgrounds, thereby introducing thus posing challenges to MCI subtype research (Berezuk et al., 2021).

The approach of defining MCI subtypes based on magnetic resonance imaging (MRI) features and data-driven methods has attracted the interest of researchers. In recent years, the extraction of features and construction of classifiers using deep learning or machine learning techniques based on MRI data have become prominent research areas. These methods have yielded promising results in AD classification and the investigation of abnormal brain connectivity patterns (Zuo et al., 2023, 2024). In subtype studies, the uncertainty of subtype categories often necessitates the use of unsupervised or semi-supervised methods. However, minor differences among MCI subjects can limit the effectiveness of simple clustering for subtype definition. Some studies have categorized MCI into subtypes that align closely with AD (e.g., A-CI, MCI-AD) or normal aging (e.g., N-CI, MCI-CN), potentially overlooking other subtypes (Kwak et al., 2021; Zhao et al., 2022). To augment the effectiveness of unsupervised clustering methods, some studies have integrated cerebrospinal fluid (CSF) markers to form clusters or statistical characteristics. For instance, Nettiksimmons et al. utilized 11 variables, including total brain volume, hippocampal volume, cortical thickness, and CSF, to segregate 138 baseline MCI subjects from ADNI into four subgroups. This segregation was based on disease severity and employed minimum variance and Euclidean distance measurement clustering methods (Nettiksimmons et al., 2014).

Dong et al. (2017) and Ten Kate et al. (2018) applied the definition of AD subtypes to construct MCI subtypes, confirming the feasibility of this method for several MCI subtypes. However, these studies have only limitedly explored MCI’s longitudinal development, lacking longitudinal subtype attribution and correlation analysis of conversion to AD. Edmonds et al. (2015) utilized cognitive scale scores to classify the MCI population within the ADNI into distinct subtypes: dynamic MCI, dysexecutive MCI, amnestic MCI, and cluster-derived normal MCI. Their findings affirm MCI heterogeneity from a cognitive standpoint. They suggested that about a third of the population-derived groups diagnosed as MCI may have false positives, advocating for more biological markers in future MCI groupings (Edmonds et al., 2016). While this false-positive theory is not widely accepted yet, their proposed four subtypes serve as a reference for future research. Thus, MCI subtyping based on MRI features warrants further study regarding subtype definition subtype, biomarker correlation, and subtype disease progression.

Given that MCI is typically the prodromal stage of AD, these subtypes were delineated through a semi-supervised mixture of experts (MOE) approach, leveraging cortical thickness measurements from T1-weighted MRI images to develop several support vector machine (SVM) classifiers. The subtype attribution of MCI was determined by the minimum distance from MCI subjects to each SVM’s hyperplane. Compared to AD, the longitudinal development of the disease is a unique research focus of the MCI stage, such as whether patients convert into AD and the conversion time (Nelson et al., 2021). This study found that in ADNI’s longitudinal acquisition, some MCI subjects’ tracking data were missing, potentially affecting the subtype analysis in the disease’s longitudinal development, a factor overlooked in previous studies. Hence, a relatively reasonable longitudinal data screening process was established to conduct a detailed analysis of neuropsychology and pathology in the subtypes’ longitudinal development.

## 2 Materials and methods

### 2.1 Participants and MRI processing

The data for this study were sourced from the ADNI database,^1^ accessed on August 30, 2023 (Mueller et al., 2005). Established in 2004, the ADNI project comprises a collection of MRI scans along with neuropsychological and neuropathological data. During the initial phase (ADNI-1), approximately 800 participants aged between 55 and 90 years were enrolled. This cohort included CN individuals, patients diagnosed with AD, and those with MCI. The specific criteria for participant inclusion and exclusion have been detailed by Petersen et al. (2010).

The eligibility criteria for the MCI group adhered to the NINCDS/ADRDA standards and included a mini-mental state examination (MMSE) score between 24 and 30, self-reported memory complaints, objectively measured memory loss (adjusted for education) using the Wechsler memory scale logical memory II, a clinical dementia rating (CDR) of 0.5, no significant impairments in other cognitive domains, and reasonably preserved daily living activities. For the CN group, the inclusion criteria were an MMSE score between 24 and 30 and a CDR of 0. Participants classified as having probable AD had an MMSE score ranging from 20 to 26 and a CDR of 0.5 or 1. Based on these inclusion criteria of each group in ADNI, all subjects who effectively underwent baseline MRI were included in subsequent studies. This resulted in a total of 192 AD patients, 396 MCI patients, and 188 CN participants (Table 1).

**Table 1:** Demographic and cognitive characteristics of the subjects.

Group	Subject number	Age (years)	Sex (female %)	MMSE	CDR-SB
MCI	364	75.4 ± 7.4	127 (34.9%)	27.0 ± 1.8	1.6 ± 0.9
AD	192	74.8 ± 7.3	109 (47.8%)	23.3 ± 2.0	4.3 ± 1.6
CN	228	75.9 ± 5.0	91 (47.4%)	29.1 ± 1.0	0.03 ± 0.12

MRI scans were conducted using 1.5 T scanners with the following parameters: a repetition time (TR) of 3,000 ms, an echo time (TE) of 3.55 ms, slice thickness of 1.2 mm, and a voxel size of 1.2 × 0.94 × 0.94 mm^3^ (Jack et al., 2008). All subjects adhered to a uniform MRI acquisition standard.

### 2.2 ROIs feature extraction

**Figure 1:**
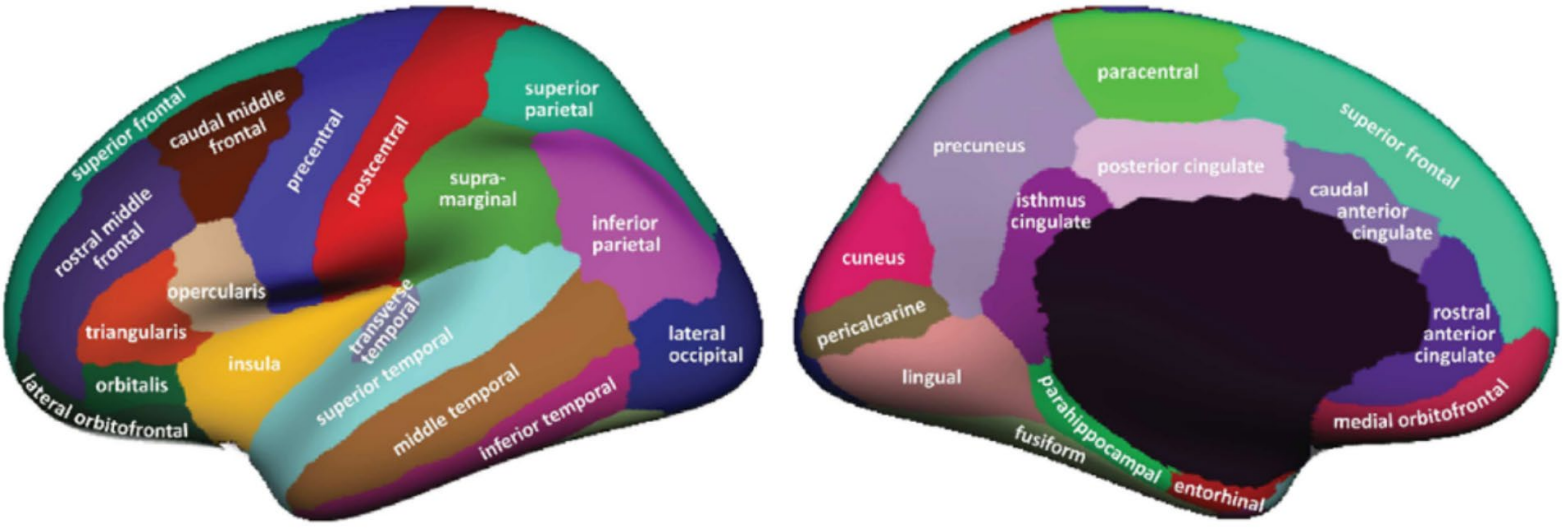
ROIs in Desikan–Killiany atlas (Desikan et al., 2006).

Cortical reconstruction and regions of interest (ROIs) segmentation for all subjects, including those with MCI, AD and CN, were performed using FreeSurfer version 4.3,^2^ accessed on August 30, 2023 (Fischl, 2012). The ADNI website supplied cortical and subcortical data on gray matter thickness using the Desikan-Killiany atlas (Desikan et al., 2006; Klein and Tourville, 2012), it divides the left and right brain into 68 ROIs (Figure 1). We use the FreeSurfer software to extract the cortical thickness of these 68 ROIs. These data were then used for further MOE subtype definition and analysis.

### 2.3 Cognitive scales and neuropathological data

The used neuropsychological data were grouped into three categories: global cognitive scales, Functional Activities Questionnaire (FAQ), and ADNI-composite scores. The first group included assessments like MMSE, CDR-Sum of Boxes (CDR-SB), and AD assessment scale-cognitive subscale (ADAS-Cog). The latter group comprised four specific sub-domains: memory, executive function, language, and visuospatial abilities. Gibbons et al. used item response theory techniques to develop composite scores for memory (ADNI-MEM), executive function (ADNI-EF), composite scores for language (ADNI-LAN) and visuospatial abilities (ADNI-*VS*) using methods comparable to the ADNI neuropsychological battery (Shaw et al., 2011; Gibbons et al., 2012; Choi et al., 2020).

All study subjects had apolipoprotein E (APOE) status information, which includes two alleles. Additionally, CSF data was collected from about half of the participants. Of the 228 CN participants, CSF data was available for 111. For the 192 AD participants, CSF beta-amyloid 1–42 (Aβ_1-42_) and phosphorylated tau (p-tau) data were available for 98 participants, while CSF total tau (t-tau) data was available for 96 (Shaw et al., 2009, 2011).

### 2.4 Definition of MCI subtype using MOE

In defining subtypes, we utilized the MOE semi-supervised method proposed by Eavani et al. (2016), which combines multiple linear SVMs with unsupervised Fuzzy-C-means (FCM). The choice to not directly use SVMs to differentiate between MCI and CN groups is due to the subtle differences between these cohorts, which lead to relatively low classification accuracy. Similarly, the definition of MCI subtypes was approached using the AD-to-MCI mapping method, following the methodologies of Dong et al. (2017) and Ten Kate et al. (2018).

Each participant received a binary label, *y*_*i*_∈ {−1,1}. The control group (CN participants) was taken as the “anchor,” and labeled as −1, while the AD patients were labeled as 1. In equation (1), *K* is the expert count, *m* is the membership value, *n* is the total AD subject count, *C* is the loss penalty, and *t* is the SVMs and FCM balancing trade-off parameter. Parameters *C* and *t* were jointly optimized using a grid search approach, with a search range from 2^−3^ to 2^10^, respectively. We used 10-fold cross-validation for the MOE. Parameters selection mainly depended on cross-validated accuracy (*Acc*), maximum pair-wise inner-product (*W_r_*), and Bezdek partition coefficient (BPC; Dave, 1996).


(1) $$\begin{array}{c} {\text{minimize}}_{{\left(w^{k}\right)}_{k}{\left\{m_{i}^{k}\right\}}_{i,k}}\sum_{k=1}^{K}\left\{\begin{array}{l} \frac{1}{2}{\left\|w^{k}\right\|}_{1}+C\sum_{i=1}^{N}m_{i}^{k}{\left(1-y_{i}{\left(w^{k}\right)}^{T}x_{i}\right)}^{2}\\ +t\sum_{i=1}^{N}{\left(m_{i}^{k}\right)}^{2}{\left\|x_{i}-d^{k}\right\|}_{F}^{2} \end{array}\right\}\\ \begin{array}{l} \text{subject to}\;\sum_{k=1}^{K}m_{i}^{k}=1,m_{i}^{k}\in \left[0,1\right].\;n=1,\cdots ,\\ N,d^{k}=\frac{\sum_{i=1}^{N}{\left(m_{i}^{k}\right)}^{\alpha }x^{i}}{\sum_{i=1}^{N}{\left(m_{i}^{k}\right)}^{\alpha }} \end{array} \end{array}$$

**Figure 2:**
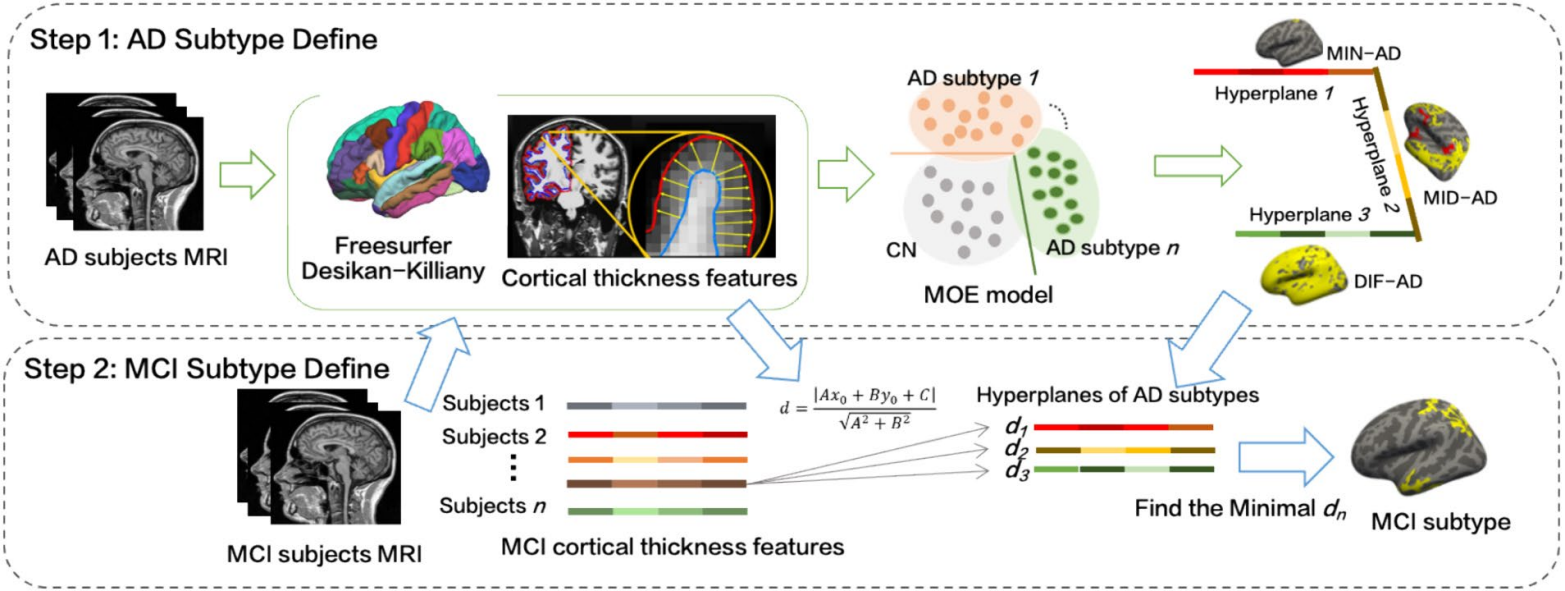
The process of MCI subtypes definition.

The specific implementation process is as follows (Figure 2):

1. The cortical thickness values of a total of 68 brain regions in the left and right cerebral hemispheres of the Desikan–Killiany atlas were obtained. To account for individual variability, factors such as age, sex, years of education, and intracranial volume (ICV) were considered extraneous variables (Sun et al., 2019). For each feature, the regression coefficient for cognitively normal controls ($\beta_{CN}$) was calculated using a generalized linear model (GLM) as equation (2):
  (2) $$\begin{array}{l} {\text{thickness\_value}}_{CN}=\beta_{CN}\times \\ \left(1+{\text{age}}_{CN}+sex_{CN}+edu_{CN}+ICV_{CN}\right)+\epsilon \end{array}$$

As shown in equation (3), then the effects of age, sex, years of education and ICV were regressed out of all subjects (MCI, AD and CN):


(3) $$\begin{array}{l} {\text{residual}}_{ALL}={\text{thickness\_value}}_{ALL}-\beta_{CN}\times \\ \left(1+{\text{age}}_{ALL}+sex_{ALL}+edu_{CN}+ICV_{ALL}\right) \end{array}$$

Thus, the obtained ${\text{thickness\_value}}_{ALL}$ served as the input feature for MOE.

2. The MOE program was executed with the number of experts set to *n* = 2, 3, 4. The class-weight for AD and CN were set at 1:1, 2:1, … 5:1. Optimal *C* and *t* were determined through evaluation indices: cross-validated *Acc*, *W_r_*, and BPC. This process led to the definition of AD subtypes, and for corresponding number of experts, the SVM hyperplane equations were derived.
3. We calculated the distance from MCI’s cortical thickness features to the three SVM hyperplanes, identified the group with the smallest distance *d_min_*, and assigned MCI to this group.
4. We used Freeview, a visualization and analysis tool from the FreeSurfer software suite, to examine cortical atrophy in each MCI and AD subgroup. Using Freeview, we generated statistical graphs that illustrated the degree of cortical atrophy, enabling us to visually contrast structural changes across variouspatient groups. This analysis specifically focused on regions with significant reductions in cortical thickness. We then conducted statistical comparisons of these measurements among the MCI, AD, and CN groups to identify areas of significant atrophy. If the cortical thickness of an ROI in CN differs from that in AD or MCI, as indicated by a *p*-value less than 0.05, it will be highlighted in yellow or red.

### 2.5 Baseline analysis of MCI subtypes

MCI subtype analysis involved neuropsychological and pathological features, including cognitive tests such as MMSE, ADAS-Cog13, CDR-SB, and executive function tests like FAQ, and ADNI composite scores. Quantitative statistical analysis of subtypes involved pairwise comparisons using the one-way analysis of variance (ANOVA) and Dunnett-t tests. The proportions of abnormal CSF and APOE carriers were determined using Chi-square tests (χ^2^), with the abnormality proportion calculated after excluding missing data.

### 2.6 Longitudinal data screen

The 24-month mark post-baseline data (M24) collection was set as the cut-off for participant screening, detailed as follows:

1. During the data screening phase, we initially screened all baseline data, MCI data for 6-month mark post-baseline (M06), MCI data for 12-month mark post-baseline (M12), MCI data for 18-month mark post-baseline (M18), and M24.
2. Referencing to the ADNI-1 acquisition protocol and description, we obtained 391 subjects in baseline. Here, we used D_bl (diagnostic baseline) indicted the disease state during the subjects’ baseline data collection.
3. Using subjects with D_bl = MCI, and M24 as the reference, data was screened in reverse order from M18, M12, to M06. For instance, M18 = (‘M24 yes’ + ‘M18 Yes’ ∩ ‘M24 No’ ∩ M36 yes) belonging to MCI (M36: 36-month mark post-baseline). This screening process was also used for M12 and M06. ‘MX Yes’ indicates the subject underwent an MRI scan at MX; ‘MX No’ signifies the absence of the subject’s MRI scan at MX.
4. To maximize subject retention, if a subject lacked tracking data at M24 but had data at M06, M12, M18, and M36, and if the M36 state was MCI, this subject would be included in MCI. However, if the M36 state was AD or CN, indicating an indeterminable transformation time, the subject would be excluded.
5. Data were sequentially screened according to the order of M06, M12, M18, and M24. Any data already been converted or reversed converted in the next period was excluded. For instance, if subjects at M06were converted to AD or reverse converted to CN, such subjects would be excluded from the M12 data and would not be included in the conversion rate for M12.

The data was screened sequentially from M06 to M24. Subjects already converted or reverse-converted in the next period were excluded. For instance, if subjects at M06 had transformed into AD or reverse-converted into CN, they would be excluded from M12 and not factored into M12’s conversion rate.

### 2.7 Longitudinal analysis of MCI subtypes

The longitudinal analysis incorporated cognitive scales like MMSE, CDR-SB, ADAS-Cog13 and ADNI composite scores. Based on the cognitive scores at M06, M12, M18, and M24 post-baseline data collection, we plotted time-dependent change curves for each scale’s score.

The longitudinal data screened in Section 2.4 were utilized in the statistical analysis of MCI conversion. The conversion rates of the three subtypes at M06, M12, M18, and M24, following baseline data acquisition were calculated, respectively. Numerous studies have indicated that APOE ε4 and APOE ε2 are pivotal factors contributing to the progression of AD (Tanzi, 2012; Belloy et al., 2023). In this study, all subjects undergoing MCI conversion were subjected to APOE analysis. Finally, the features of the converted MCI subjects who converted at the time point of AD conversion were input into the SVM hyperplane equations for AD subtype definition. The subtype with the minimum distance existed was identified, and the subtype to which each transformed subtype was attributed was calculated accordingly.

The longitudinal data from 2.5 were used to calculate MCI conversion rates for three subtypes at M06, M12, M18, and M24 post-baseline. All subjects undergoing MCI conversion were analyzed for APOE. The features of MCI subjects at the AD conversion point were input into SVM hyperplane equations for AD subtype definition. The subtype with the minimum distance was identified, and the corresponding transformed subtype was calculated.

## 3 Results

### 3.1 Atrophy patterns of MCI subtypes

**Figure 3:**
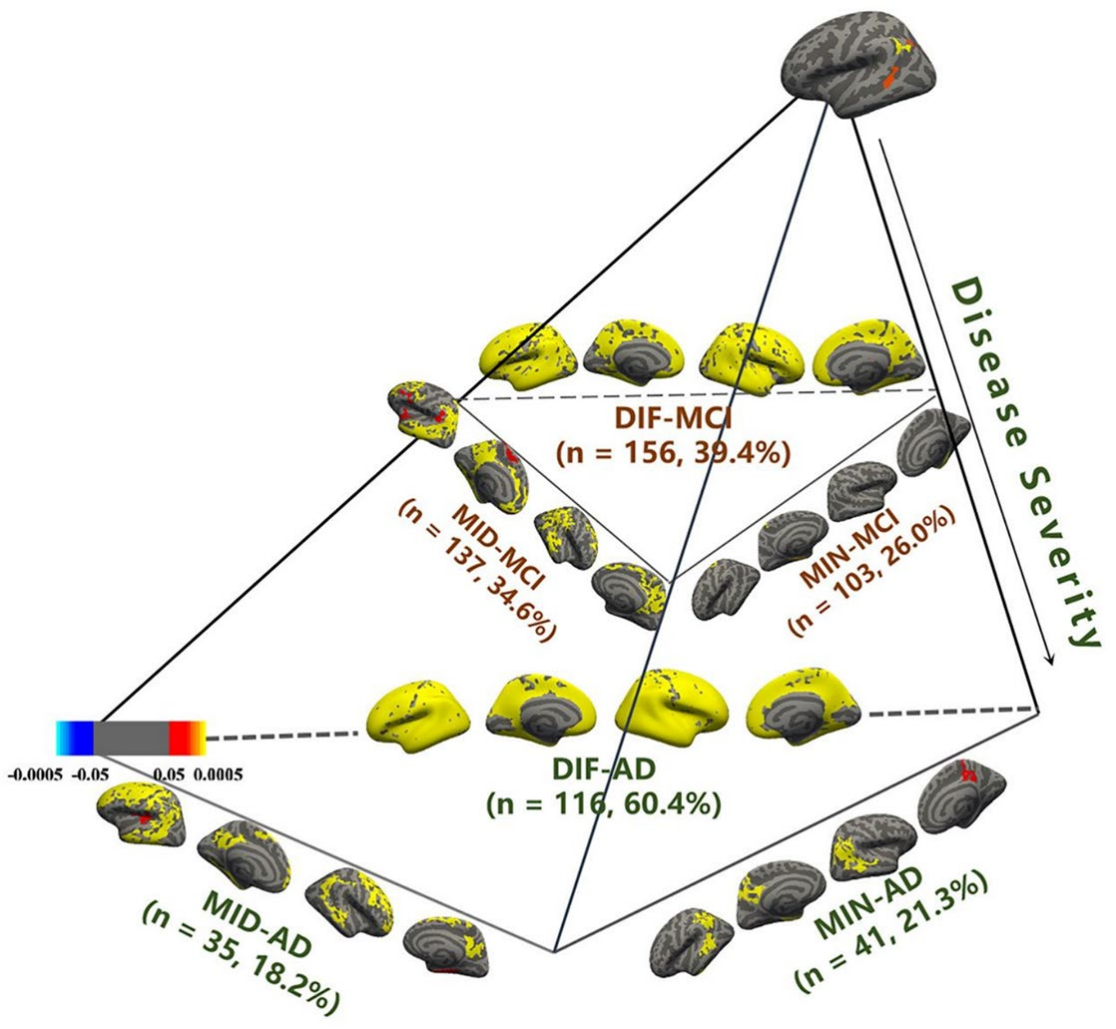
Identification of mild cognitive impairment (MCI) subtypes in comparison with cognitive normal (CN).

Statistical parameter diagrams were used to analyze the cortical thickness features of CN for each AD and MCI subtype. MCI and AD each developed distinct subtypes within three atrophy region groups. With three MOE experts, *Acc* = 85.3 ± 3.1%, *r_w_* = 0.32 ± 0.03, and BPC = 0.68 ± 0.09. Each subtype represented varying atrophy severity. Subtypes were named as minimal atrophy MCI (MIN-MCI), middle atrophy MCI (MID-MCI), and diffuse atrophy MCI (DIF-MCI) based on atrophy degree from low to high (Figure 3).

MIN-AD exhibited significant atrophy in the temporal lobe, while MIN-MCI showed almost no atrophy. In the MID group, MID-AD showed atrophy in all parts of the occipital lobe, while MID-MCI showed atrophy mainly in the temporal and frontal lobes but less in the parietal lobe. In the DIF group, both AD and MCI showed noticeable diffuse atrophy, with AD’s atrophy regions being more widespread. Regarding subtype proportions, the DIF group of AD accounted for 60.4%, whereas the DIF group of MCI was only 39.4%. The proportions in the MIN group (26.0%) and MID group (34.6%) of MCI exceeded those in the MIN (21.3%) and MID (18.2%) groups of AD (Table 1).

### 3.2 Analysis of MCI subtypes

The demographic and cognitive characteristics of three MCI subtypes and the CN group were compared (Table 2). The DIF-MCI subtype, which exhibited the highest degree of cortical atrophy, was significantly different in age compared to the other two subtypes. The cognitive performance of the MIN-MCI subtype was markedly superior to the other two subtypes. Despite the differences in the degree of atrophy, the cognitive evaluation differences between MID-MCI and DIF-MCI were not substantial (*p* > 0.05). As per Table 3, the proportion of APOE ε2 carriers was highest in the MIN-MCI subtype, while the proportion of APOE ε4 carriers was lowest. No significant differences were observed between MCI subtypes in terms of CSF markers, including Aβ_1-42_, t-tau and p-tau. Notably, nearly half of the MCI subjects did not participate in the CSF data collection. Data screening and conversion analysis.

**Table 2:** Demographic and cognitive characteristics of the subtypes.

Characteristic	CN	MCI	MIN-MCI	MID-MCI	DIF-MCI	*p*-value
*n* (%)	228	396	103 (26.0%)	137 (34.6%)	156 (39.4%)	
Age (years)	75.9 ± 5.0	74.7 ± 7.4	73.1 ± 7.5	73.1 ± 8.0	76.7 ± 6.3	<0.001*^b,c^*
Female, *n* (%)	109 (47.8%)	141 (35.6%)	34 (33.0%)	51 (37.2%)	56 (35.9%)	0.485*^d^*
Education (years)	16.1 ± 2.9	15.6 ± 3.0	16.0 ± 2.8	15.2 ± 3.0	15.6 ± 3.2	0.269
MMSE	29.1 ± 1.0	27.0 ± 1.8	27.3 ± 1.7	26.7 ± 2.0	27.0 ± 1.8	0.137
CDR-SB	0.03 ± 0.12	1.6 ± 0.9	1.5 ± 0.8	1.5 ± 0.8	1.7 ± 1.0	0.475
FAQ	0.14 ± 0.6	3.8 ± 4.5	3.3 ± 4.2	3.6 ± 4.3	4.3 ± 4.7	0.141
ADAS-Cog 13	9.5 ± 4.2	18.6 ± 6.3	17.6 ± 5.8	19.5 ± 6.5	18.3 ± 6.3	0.047*^a^*
ADNI-MEM	0.97 ± 0.53	−0.08 ± 0.58	−0.04 ± 0.58	−0.20 ± 0.55	−0.07 ± 0.60	0.005*^a,c^*
ADNI-EF	0.64 ± 0.75	−0.04 ± 0.86	−0.22 ± 0.74	−0.10 ± 0.85	−0.15 ± 0.91	0.001*^a,b^*
ADNI-LAN	0.78 ± 0.75	−0.06 ± 0.76	−0.06 ± 0.71	−0.07 ± 0.76	−0.12 ± 0.79	0.209
ADNI-*VS*	0.23 ± 0.60	−0.13 ± 0.79	−0.04 ± 0.72	−0.13 ± 0.79	−0.23 ± 0.81	0.019*^b^*

^a^Significant differences (*p* < 0.05) between MIN-MCI and MID-MCI; ^b^significant differences (*p* < 0.05) between MIN-MCI and DIF-MCI; ^c^significant differences (*p* < 0.05) between MID-MCI and DIF-MCI; ^d^χ^2^ test was used.

**Table 3:** Neuropathological characteristics of the subtypes.

Characteristic	CN	MCI	MIN-MCI	MID-MCI	DIF-MCI	*p*-value
APOE ε4 [*n* (carry %)]	60 (26.3%)	211 (53.3%)	48 (46.6%)	77 (56.2%)	86 (55.1%)	0.654
1	55 (24.1%)	164 (41.4%)	39 (37.9%)	57 (41.6%)	68 (43.6%)	0.578
2	5 (2.1%)	47 (11.9%)	9 (8.7%)	20 (14.6%)	18 (11.5%)	0.032*^a,c^*
APOE ε2 [*n* (carry %)]	21 (9.2%)	29 (7.3%)	11 (10.7%)	9 (6.6%)	9 (5.8%)	0.012*^a,b,c^*
Aβ_1-42_ (ng/L)	205.8 ± 54.7	81.9 ± 55.1	85.4 ± 60.5	87.4 ± 53.2	71.2 ± 53.4	0.787
Aβ_1-42_ (abnormal %)	44 (37.6%)	147 ± 75.3%	37 (71.2%)	58 (77.3%)	54 (76%)	0.170
*n* missing of Aβ_1-42_ (%)	111 (48.7%)	198 (50%)	51 (49.5%)	62 (45.3%)	85 (54.5%)	
t-tau (ng/L)	69.7 ± 29.8	51.4 ± 60.8	47.4 ± 45.4	56.4 ± 62.9	49.1 ± 67.8	0.639
t-tau (abnormal %)	21 (17.6%)	88 (45.1%)	22 (44.0%)	31 (41.3%)	34 (47.9%)	0.727
*n* missing of t-tau (%)	109 (47.8%)	201 (50.8%)	53 (51.5%)	62 (45.3%)	85 (55.1%)	
p-tau (ng/L)	25.1 ± 14.6	17.8 ± 18.0	17.5 ± 16.6	18.9 ± 16.4	16.9 ± 20.6	0.859
p-tau (abnormal %)	42 (35.3%)	140 (70.4%)	39 (75.0%)	53 (69.7%)	48 (67.6%)	0.320
*n* missing of p-tau (%)	94 (41.2%)	197 (49.7%)	51 (49.5%)	61 (44.5%)	85 (55.1%)	

*^a^*Significant differences (*p* < 0.05) between MIN-MCI and MID-MCI; ^b^significant differences (*p* < 0.05) between MIN-MCI and DIF-MCI; ^c^χ^2^ test was used. All subjects with APOE ε4 and APOE ε2 were tested.

#### 3.2.1 Longitudinal data screening

**Figure 4:**
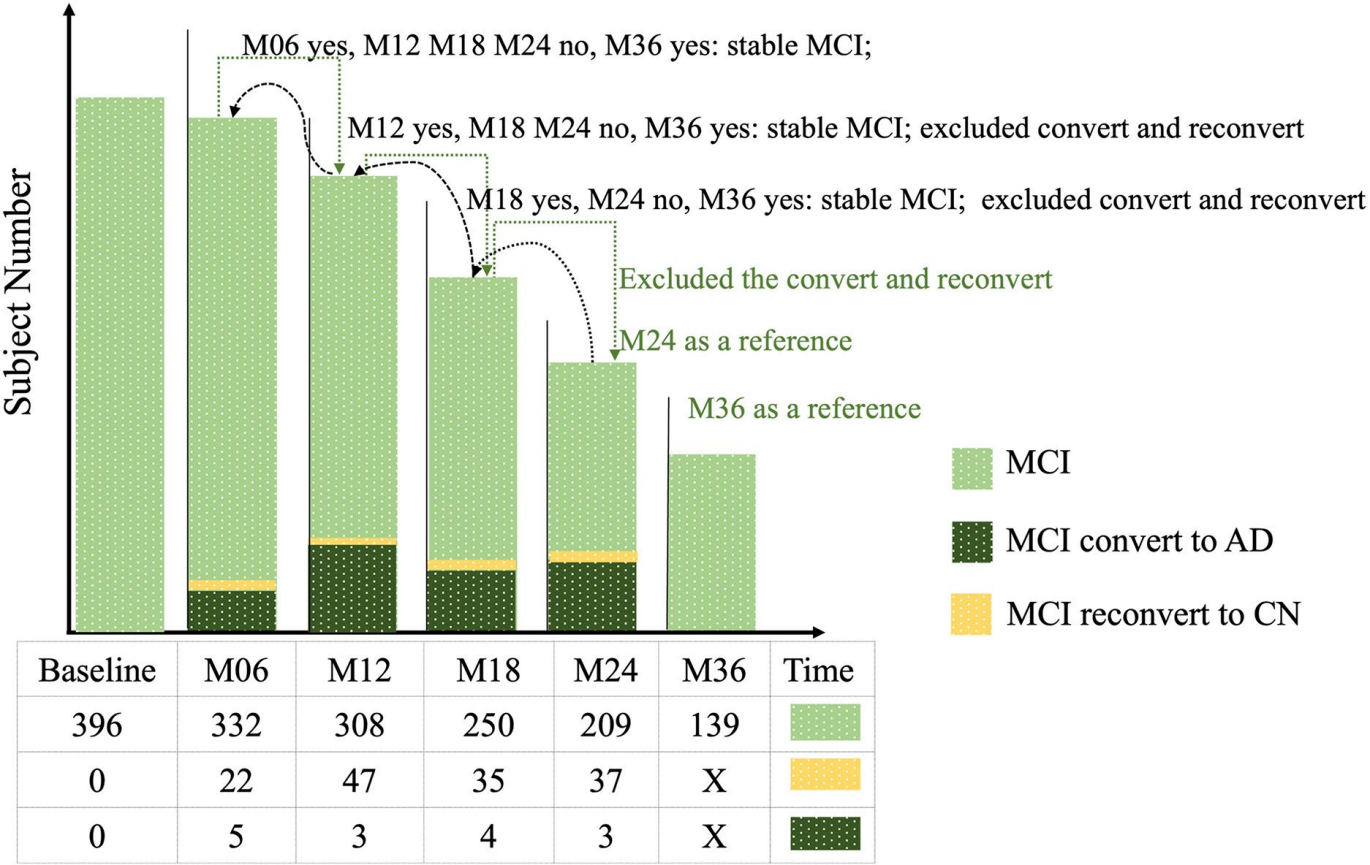
Longitudinal mild cognitive impairment (MCI) data screening.

**Figure 5:**
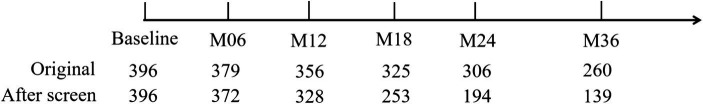
Comparison of the numbers of different data screening methods.

**Figure 6:**
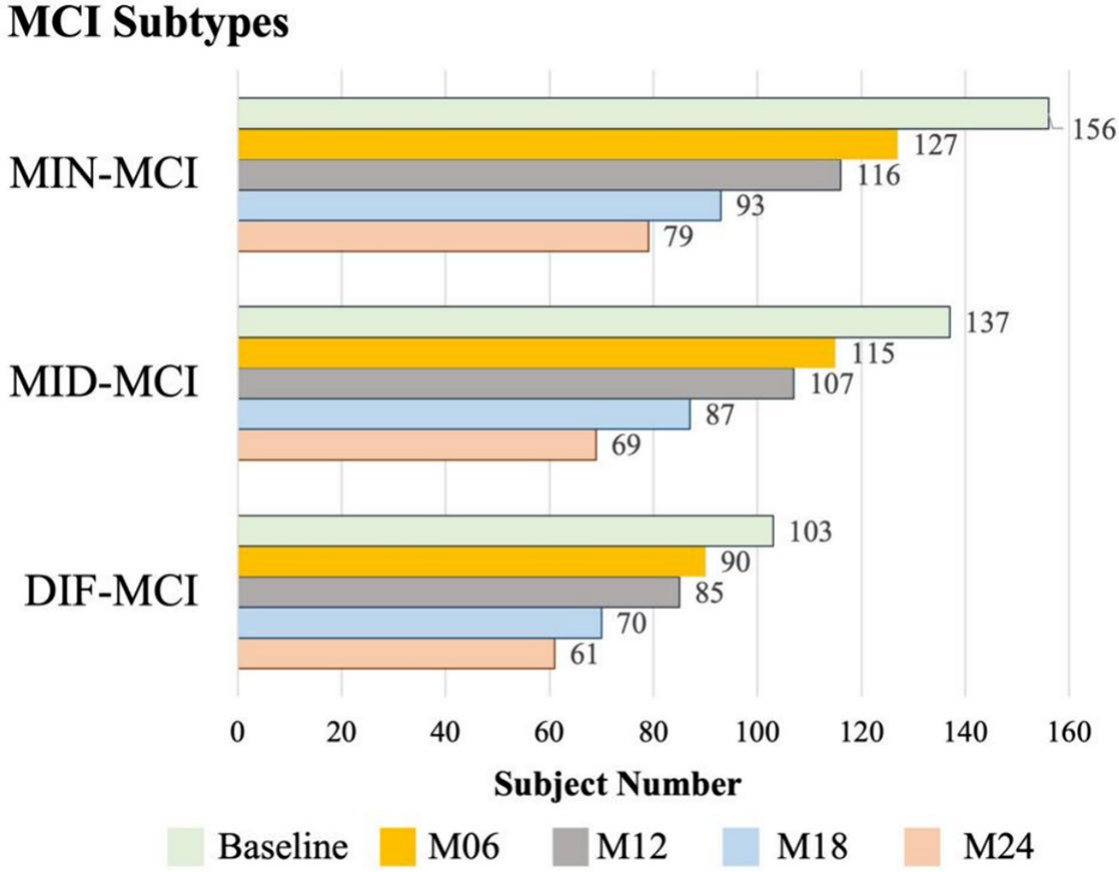
Statistics of longitudinal subject numbers for each subtype.

The initial dataset for MCI in the ADNI-1 stage comprised T1-weighted MRI data, with the original subject count recorded. Subsequently, all longitudinal data underwent screening based on the criteria outlined in Section 2.5. This screening process involved assessing subjects at each acquisition time point and identifying those who experienced subtype conversion. The subject numbers resulting from this screening process are displayed in Figure 4. Furthermore, the original data and the screened data were statistically compared, as illustrated in Figure 5. Notably, as the acquisition time extended, the discrepancy between the subject counts calculated by the two statistical methods gradually widened. By adhering to the rules outlined in this study and employing quantitative tracking data for each subtype, MID-MCI achieved the relative minimum atrophy degree. At the 24-month follow-up (M24) after baseline, approximately 60% of the subjects initially included in the baseline data acquisition were retained, with a similar proportion observed in the other two subtypes (Figure 6).

#### 3.2.2 Analysis of longitudinal data conversion

**Figure 7:**
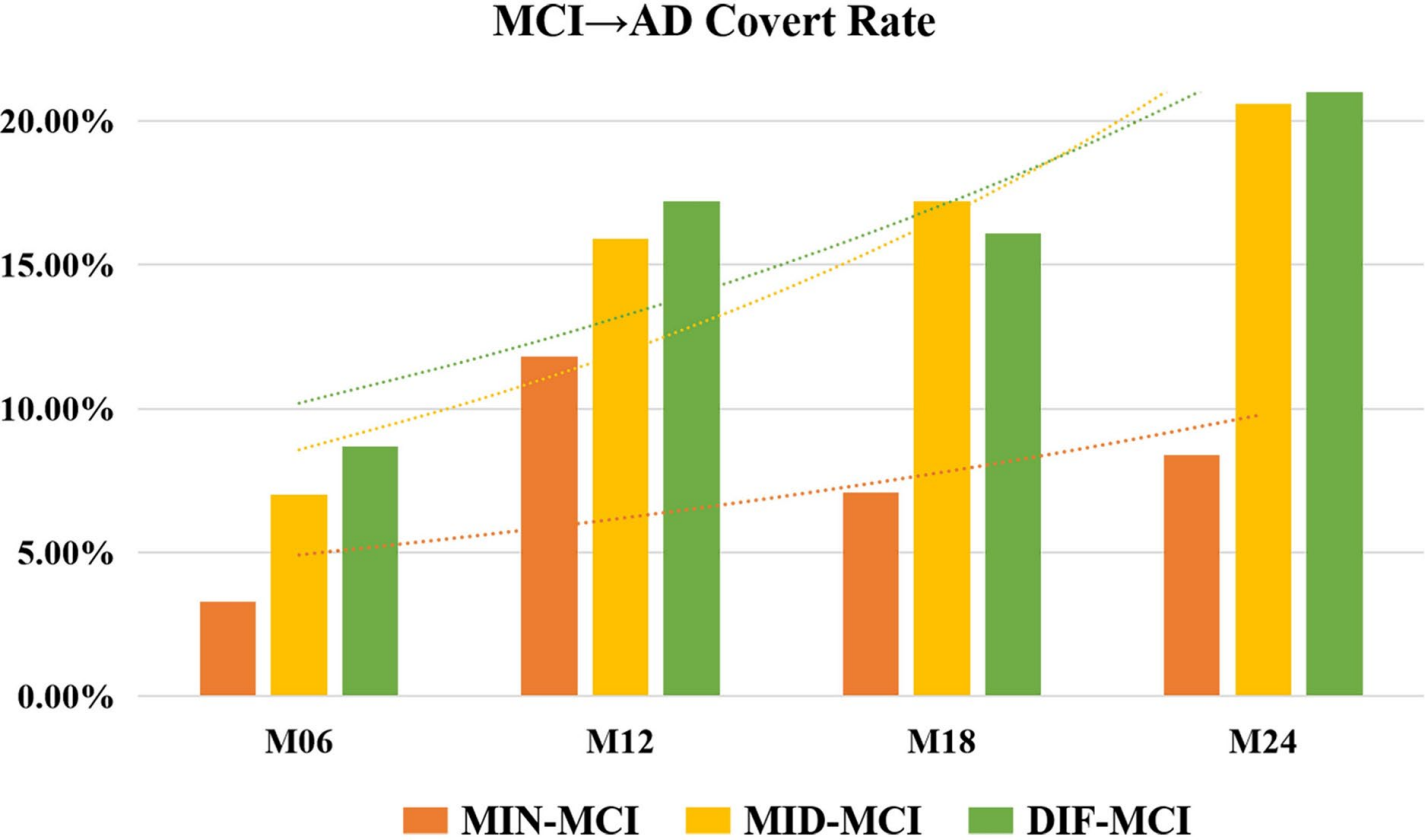
Rate of mild cognitive impairment (MCI) conversion to Alzheimer’s disease (AD) for each subtype.

**Figure 8:**
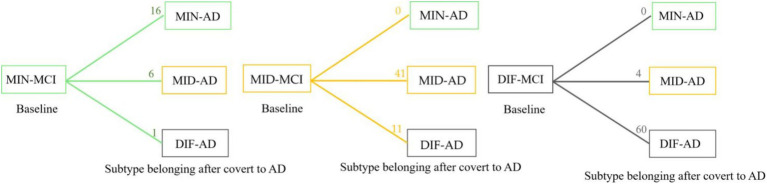
Statistics of longitudinal numbers for each subtype. The number on each line represents the number of subjects who converted from mild cognitive impairment (MCI) to Alzheimer’s disease (AD); the subtype has transformed or remained itself.

In a comprehensive analysis of M24 data, the incidence of MCI conversion to AD was examined across three subtypes: MIN-MCI: 23 subjects (M06: 3, M12: 10, M18: 5, M24: 5); MID-MCI: 52 subjects (M06: 8, M12: 17, M18: 15, M24: 12); DIF-MCI: 64 subjects (M06: 11, M12: 20, M18: 15, M24: 18). Among these subtypes, the conversion rate for MIN-MCI was the lowest, while that of MID-MCI surpassed that of DIF-MCI by the time M18 was reached. After M18, the conversion rates of the MID-MCI and DIF-MCI were similar (Figure 7). Notably, when considering APOE carriers among MCI subjects who transitioned to AD, it was observed that the proportion of APOE ε2 carriers within each subtype remained below 5%, whereas the prevalence of APOE ε4 carriers exceeded 60% (Table 4). Figure 8 illustrates the subtypes associated with MCI subjects who converted to AD, revealing that a majority of subjects were classified into the AD subtype corresponding to their baseline MCI status, thus maintaining a consistent attribution to the same disease group.

**Table 4:** Apolipoprotein E (APOE) carries of subjects converted to Alzheimer’s disease (AD).

APOE	MIN-MCI	MID-MCI	DIF-MCI
APOE ε2 (carry %)	1 (4.3%)	1 (1.9%)	3 (4.6%)
APOE ε4 (carry %)	17 (73.9%)	35 (67.3%)	42 (65.6%)

### 3.3 Cognitive and demographic characteristics of longitudinal

**Figure 9:**
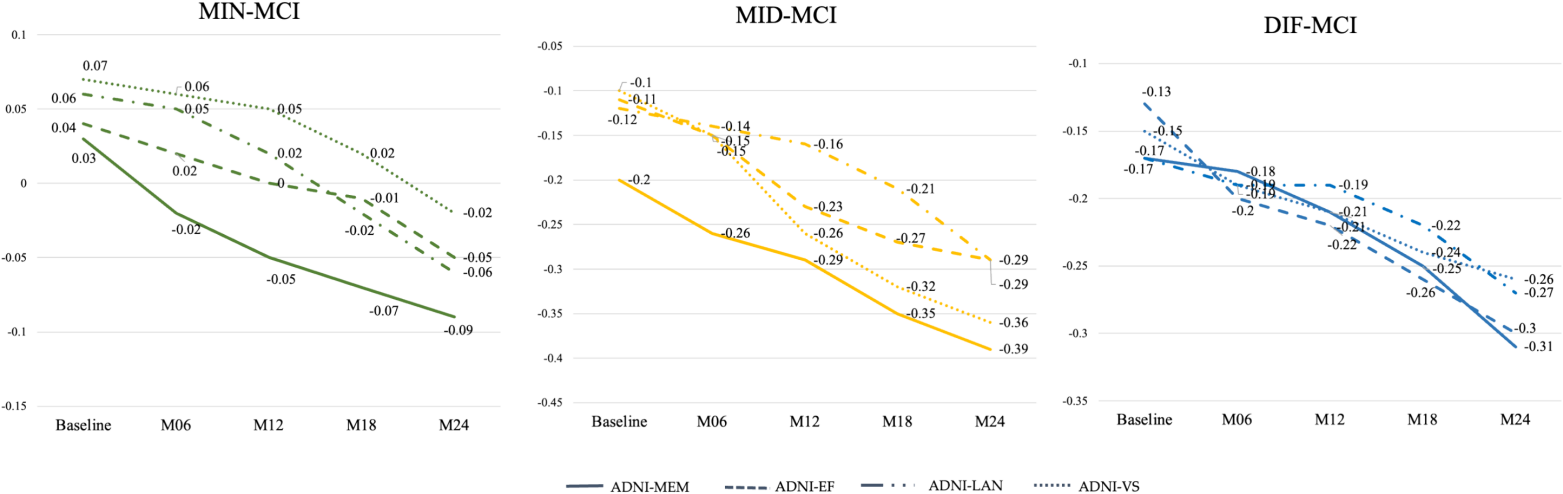
Longitudinal cognitive changes in ADNI-composite scores.

**Figure 10:**
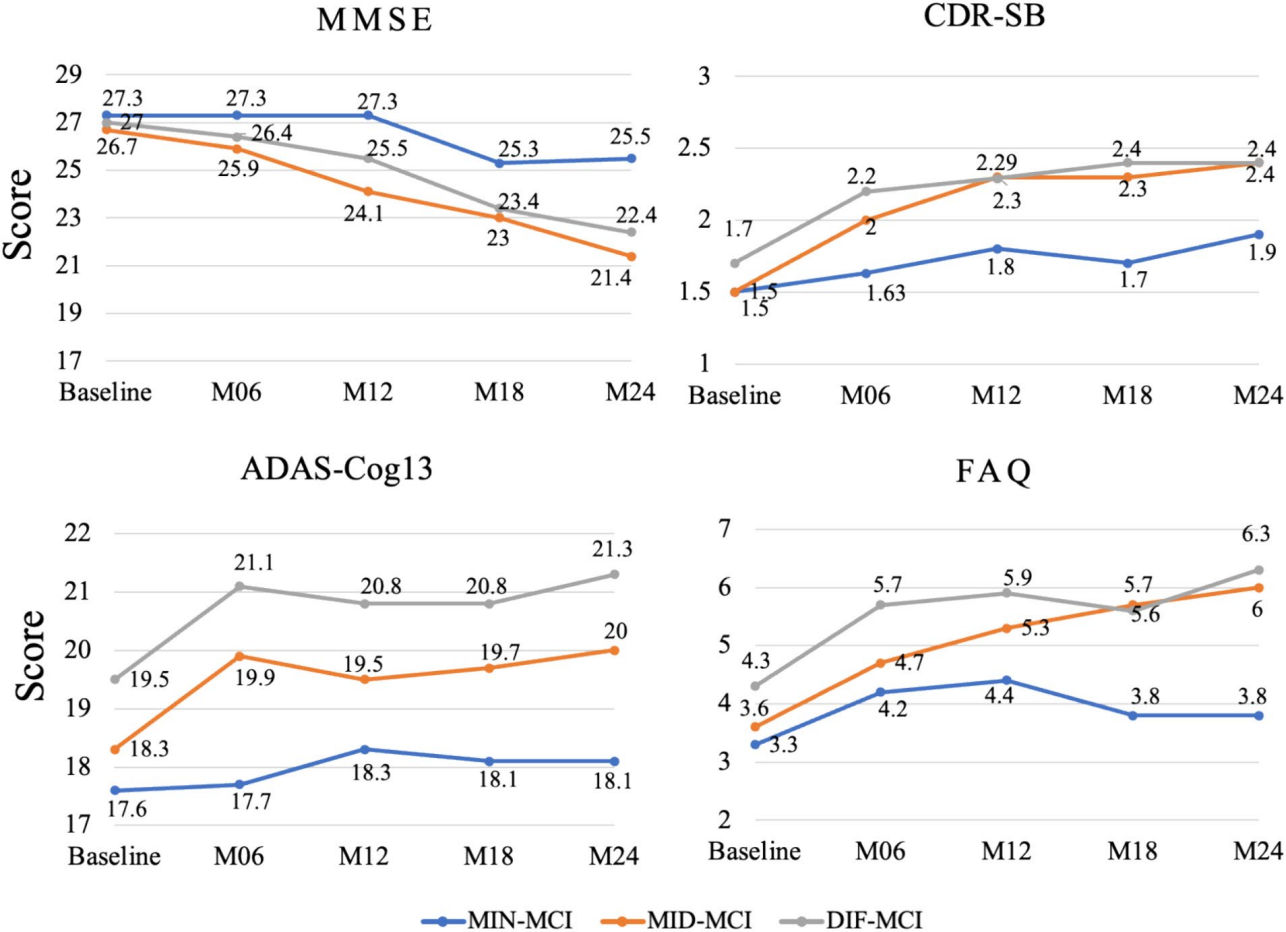
Longitudinal cognitive changes in global assessments and FAQ.

Based on the average cognitive scores of all subjects in each subtype, from Figures 9, 10, we can observe that over time, the cognitive scores of MID-MCI in various tests, including ADNI-MEM, ADNI-EF, and ADNI-*VS*, exhibited a more rapid decline compared to those of DIF-MCI (Figure 9). Specifically, the MMSE score for MID-MCI (from 26.7 in baseline to 21.4 in M24) was lower than that of DIF-MCI (from 27.0 in baseline to 22.4 in M24). On the other hand, the longitudinal tracking data for MIN-MCI in FAQ and ADAS showed a gradual decline, occasionally fluctuating, without any pronounced downward trend over a two-year period (Figure 10).

## 4 Discussion

This study, based on the cortical thickness features of 396 MCI patients from the ADNI-1 database, defined and analyzed three subtypes of cortical atrophy in MCI. It includes the definition and analysis of baseline MCI subtypes, the developmental trajectories of MCI over 2 years, and the APOE analysis of MCI subjects who converted to AD. In addition, a data-filtering scheme for longitudinal studies from ADNI was proposed. The key findings are as follows: First, when examining atrophy patterns, nearly one-third of the MCI subjects showed almost no significant difference in cerebral cortex thickness compared with CN individuals. These subjects exhibited a slower cognitive decline and a lower conversion rate to AD. Second, the MID-MCI subtype, characterized by significant atrophy in the temporal and frontal lobes, displayed a faster decline rate in various cognitive performances compared with the DIF-MCI subtype. It was also discovered that APOE ε4 was a significant factor influencing whether MCI converts to AD. Upon exploring ADNI tracking data, it was observed that different methods of selecting data from the ADNI database for longitudinal tracking resulted in significant variations in data quantity. This study proposes a more comprehensive selection method. For future studies involving longitudinal data on MCI, it is recommended to select longitudinal data according to the experimental design plan. These findings offer valuable insights into enhancing clinical progression prediction and addressing the classification accuracy of MCI.

### 4.1 Characteristics analysis of MCI subtypes

Following the definition of subtypes, three distinct AD subtypes emerged, each characterized by specific cerebral atrophy features, all prominently affecting the temporal lobe. Among AD patients with memory disorders, amnestic MCI plays a pivotal role in the early stages. Notably, the temporal lobe cortex is closely associated with memory function. In the corresponding MCI subtypes, approximately 26% of subjects exhibited minimal atrophy in the lateral temporal lobe, while only a minority experienced atrophy in the medial temporal lobe. These findings suggest that, in terms of cognitive measurement, all such subjects displayed similar MCI symptoms. Morphologically, this group of subjects leaned more toward CN than typical AD. Previous studies have consistently demonstrated that lateral temporal lobe atrophy is a key discriminator between AD and MCI, whereas thinning of the medial temporal lobe is associated with the difference between CN and MCI. MCI, positioned between AD and CN, exhibits a nuanced spectrum. The study results underscore that this particular group of MCI subjects tends to align more closely with the CN category (Dubois and Albert, 2004; Bondi et al., 2014).

When comparing the three MCI subtypes, MIN-MCI stands out with a relatively low proportion of APOE ε4 carriers, while the incidence of APOE ε2 carriers is notably higher (though still lower than in CN subjects). Additionally, an abnormal proportion of tau protein is observed in this subtype. The underlying reasons are multifaceted. First, the APOE ε4 genetic factor contributes to the abnormal accumulation of pathological markers like tau, thereby triggering cognitive decline. Second, the APOE ε2 genetic factor appears to exert a protective effect on the cerebral cortex, effectively slowing down cortical atrophy. However, in our study, the carrier rate of APOE ε2 was less than 5%, indicating that it is not sufficient to conclude that APOE ε2 plays a significant role in protecting the cerebral cortex. One notable limitation that cannot be ignored is that during the data collection phase of ADNI-1, CSF data for tau and Aβ_1-42_ were available for only half of the subjects, which means that our conclusions still require further experimental data for future confirmation.

Regarding the subtype MID-MCI, atrophy predominantly appeared in the frontal lobe and temporal lobe. From the observational data in Figure 10, it can be seen that although the degree of atrophy was not as pronounced as that observed in DIF-MCI, MID-MCI exhibited the poorest performance in certain cognitive domains, such as the MMSE. Additionally, MID-MCI had the highest proportion of APOE ε4 carriers and abnormalities in Aβ_1-42_ levels, along with the fastest decline during multiple longitudinal cognitive tests. By M18, the proportion of subjects transitioning to AD surpassed that of DIF-MCI. Among the MCI subtypes defined by Whitwell et al. (2007) using cognitive measurements, multidomain MCI subgroups were characterized by injury to the temporal lobe, cingulate cortex, and prefrontal lobe. Longitudinal studies have highlighted that this type of gray matter atrophy is common among individuals who later progress to AD (Whitwell et al., 2008).

The atrophy regions observed in DIF-MCI closely resembled those found in diffuse AD. Notably, a higher proportion of subjects from the DIF-MCI group transitioned to AD compared to the other two subtypes. Researchers, including Edmonds et al. (2016), Winblad et al. (2004), and Petersen and Morris (2005) have identified a similar subtype referred to as “Multidomain amnestic MCI.” Despite the absence of pronounced cortical atrophy over several years, this subtype continues to exhibit a high transformation rate, indicating an elevated pathogenic risk for AD.

In the realm of cognitive assessment, the MMSE score for DIF-MCI surpassed that of MID-MCI, indicating better cognitive performance in the former. However, the FAQ decline rate in MID-MCI was the most rapid among the three subtypes. Notably, the ADNI has not provided an estimation of the disease course for MCI, which remains a current research challenge in the field of MCI and AD. Based on the findings of this study, it is speculated that most MID-MCI subjects are in a rapid progression stage relative to the other two subtypes. Furthermore, while the MMSE is widely used as an AD measurement method, the study results suggest that it may be more applicable for classifying AD, MCI, and CN individuals rather than distinguishing the severity levels among MCI patients.

The analysis conducted in our research indicates that the established subtypes of MCI reflect the disease’s severity to some extent, akin to distinct courses of the disease. Specifically, the atrophy regions observed in MID-MCI resemble stages 4–5 of nerve fiber tangles according to Braak staging, whereas MIN-MCI aligns more closely with stages 1–3 (Braak et al., 2011). Our findings reveal that MIN-MCI subjects exhibit the slowest disease progression, MID-MCI subjects experience an accelerated stage, and DIF-MCI subjects exhibit relatively slow cognitive decline despite significant cortical atrophy. Additionally, an analysis of converted patients demonstrates a high proportion of APOE ε4 carriers among those progressing to AD, while APOE ε2 carriers are nearly absent. This underscores the role of APOE as a crucial pathogenic factor in MCI to AD transformation. Recently, conducted a review and analysis of disease progression, emphasizing the impact of MCI severity on various studies, including MCI conversion and classification. The method proposed in our study offers a practical and feasible reference for follow-up MCI research (Brück et al., 2021).

### 4.2 Longitudinal analysis of MCI subtypes based on the ADNI database

In the realm of longitudinal studies involving MCI subjects from the ADNI, significant variations exist in the number of subjects tracked over time (Vivek et al., 2006; Jack et al., 2010). Longitudinal research is pivotal for understanding MCI progression. However, our statistical findings reveal challenges in precisely determining disease transformation timing for certain subjects during data collection. For example, some individuals experience AD transformation during secondary data acquisition after missing the initial assessment. Additionally, certain subjects participate only in baseline data collection due to various reasons. These factors impact subsequent calculations of transformation rates for specific MCI subtypes or subgroups. In addition, only 2-year longitudinal data on some subjects is available, while the transformation rate or cognitive decline has been calculated on a 3 or 4 year basis in some studies.

Indeed, Lo et al.’s statistical analysis highlights a crucial aspect: the subjects with missing data in the ADNI are not randomly distributed (Lo and Jagust, 2012). Rather, this missing data appears to be correlated with specific subject features. These features extend beyond cognitive functions and may also involve factors like biomarkers. Different biomarkers could contribute to data gaps at various time points, and the missing data patterns differ between AD and MCI subjects. Our study’s formulated rules reveal an interesting trend: a higher proportion of MIN-MCI subjects are followed up within 2 years compared to the other two groups. This phenomenon might be attributed to the favorable cognitive status and slower disease progression observed in these subjects. Figuring out the effects of missing data is very important for designing future longitudinal AD studies and clinical tests and also provides necessary conditions for ensuring data integrity and reliability.

### 4.3 Limitations and perspectives for future research

From a data perspective, the combination of multimodal MRI can provide more comprehensive information about early-stage AD, aiding in understanding the patterns and essence of the development of MCI subtypes. In data-driven MCI and AD research, numerous studies have underscored the importance of multimodal and multifeature approaches (Pan et al., 2021; Zuo et al., 2021). Data used in this study were sourced entirely from the ADNI database. ADNI-1, which used uniform inclusion criteria for MCI and included approximately 400 subjects, was suitable for MCI research. However, ADNI-1 only collected T1-weighted MRI data for all subjects and excluded DTI and fMRI data for all participants. While ADNI-2 could provide multimodal MRI data, MCI was divided into early MCI and late MCI groups, with each group having fewer than 200 subjects, not meeting the sample requirements of this study. Therefore, this study only used T1-weighted MRI data. Future research will necessitate the integration of more clinically relevant multimodal MRI data for MCI research to better elucidate the essence of different subtypes.

From an algorithmic standpoint, this study employed a semi-supervised mixed-expert algorithm MOE. Semi-supervised algorithms are capable of simulating a spectrum of changes from normal aging to disease in a 1-to-*k* form, offering a more rational and interpretable approach compared with standalone clustering methods. The integration of SVM and FCM in this study facilitated rapid algorithm efficiency, and utilized multiple classification boundaries of SVM to determine the subtype attribution of MCI samples. However, there are areas that require further improvement. For instance, when dealing with imbalanced data, we assigned larger weight values to the AD category samples, which may sometimes lead to overfitting and necessitate repeated attempts. Future research could explore the use of generative adversarial networks (GAN) to generate reconstructed images and other methods to address class imbalances more effectively (Hu et al., 2020). Although the current state-of-the-art work involving the combination of GAN networks with clustering has proven effective in defining AD subtypes, additional research is needed to verify whether this approach can effectively map AD results to MCI subtypes (Yang et al., 2021). Moreover, while research using data-driven methods in neuroimaging contributes to the understanding of the complexity of disease subtypes, the diversity of discovery research methods, robustness, reproducibility, and clinical relevance of clustering algorithms still require further validation and improvement (Chen et al., 2023).

## 5 Conclusion

In this study, a semi-supervised algorithm was employed to investigate cortical thickness features in 396 patients with MCI using the ADNI-1 database. It was found that the cerebral cortex of about 1/3 of the MCI patients was not significantly different from CN, accompanied by a low cognitive decline and a low transformation rate to AD. Conversely, MCI patients with significant atrophy in the temporal lobe and frontal lobe demonstrated a higher cognitive decline rate compared to other subtypes. Notably, the APOE ε4 gene variants were identified as critical factors influencing the progression from MCI to AD.

Furthermore, the researchers proposed a data screening method specifically tailored for the longitudinal analysis of ADNI data, aimed at enhancing data accuracy and reliability. Their investigation yields crucial insights into comprehending the progression of MCI and predicting its transition to AD. These research findings are not only enlightening for clinical intervention and treatment but also provide a new research direction.

## Data availability statement

Publicly available datasets were analyzed in this study. Data used in the preparation of this article were obtained from the Alzheimer’s Disease Neuroimaging Initiative (ADNI) database (adni.loni.usc.edu).

## Ethics statement

The studies involving humans were approved by Alzheimer’s Disease Neuroimaging Initiative. The studies were conducted in accordance with the local legislation and institutional requirements. The participants provided their written informed consent to participate in this study.

## Author contributions

BZ: Conceptualization, Data curation, Formal analysis, Funding acquisition, Investigation, Methodology, Software, Writing – original draft, Writing – review & editing. MX: Conceptualization, Data curation, Formal analysis, Investigation, Methodology, Software, Writing – original draft, Writing – review & editing. QW: Formal analysis, Investigation, Validation, Writing – review & editing. SY: Data curation, Investigation, Methodology, Validation, Writing – review & editing. YZ: Conceptualization, Supervision, Validation, Visualization, Writing – review & editing. ZL: Conceptualization, Methodology, Project administration, Supervision, Visualization, Writing – review & editing.

## Footnotes

^1^ www.adni.loni.usc.edu

^2^ http://freesurfer.net/

## References

[ref1] Belloy M. E. Andrews S. J. le Guen Y. Cuccaro M. Farrer L. A. Napolioni V.. (2023). APOE genotype and Alzheimer disease risk across age, sex, and population ancestry. JAMA Neurol. 80, 1284–1294. doi: 10.1001/jamaneurol.2023.3599, PMID: 37930705 PMC10628838

[ref2] Berezuk C. Scott S. C. Black S. E. Zakzanis K. K. (2021). Cognitive reserve, cognition, and real-world functioning in MCI: a systematic review and meta-analysis. J. Clin. Exp. Neuropsychol. 43, 991–1005. doi: 10.1080/13803395.2022.2047160, PMID: 35365060

[ref3] Bondi M. W. Edmonds E. C. Jak A. J. Clark L. R. Delano-Wood L. McDonald C. R.. (2014). Neuropsychological criteria for mild cognitive impairment improves diagnostic precision, biomarker associations, and progression rates. J. Alzheimers Dis. 42, 275–289. doi: 10.3233/JAD-140276, PMID: 24844687 PMC4133291

[ref4] Braak H. Thal D. R. Ghebremedhin E. Tredici K. D. (2011). Stages of the pathologic process in Alzheimer disease: age categories from 1 to 100 years. J. Neuropathol. Exp. Neurol. 70, 960–969. doi: 10.1097/NEN.0b013e318232a379 22002422

[ref5] Brück C. C. Wolters F. J. Ikram M. A. de Kok I. M. (2021). Heterogeneity in reports of dementia disease duration and severity: a review of the literature. J. Alzheimers Dis. 84, 1515–1522. doi: 10.3233/JAD-210544, PMID: 34690139 PMC8764595

[ref6] Chen P. Zhang S. Zhao K. Kang X. Rittman T. Liu Y. (2023). Robustly uncovering the heterogeneity of neurodegenerative disease by using data-driven subtyping in neuroimaging: a review. Brain Res. 1823:148675. doi: 10.1016/j.brainres.2023.148675 37979603

[ref7] Choi S. E. Mukherjee S. Gibbons L. E. Sanders R. E. Jones R. N. Tommet D.. (2020). Development and validation of language and visuospatial composite scores in ADNI. Alzheimer’s & Dementia: Translational Res. Clinical Interventions 6:e12072. doi: 10.1002/trc2.12072, PMID: 33313380 PMC7718716

[ref8] Dave R. N. (1996). Validating fuzzy partitions obtained through c-shells clustering. Pattern Recogn. Lett. 17, 613–623. doi: 10.1016/0167-8655(96)00026-8

[ref9] Desikan R. S. Ségonne F. Fischl B. Quinn B. T. Dickerson B. C. Blacker D.. (2006). An automated labeling system for subdividing the human cerebral cortex on MRI scans into gyral based regions of interest. NeuroImage 31, 968–980. doi: 10.1016/j.neuroimage.2006.01.021, PMID: 16530430

[ref10] Dong A. Toledo J. B. Honnorat N. Doshi J. Varol E. Sotiras A.. (2017). Heterogeneity of neuroanatomical patterns in prodromal Alzheimer’s disease: links to cognition, progression and biomarkers. Brain 140, 735–747. doi: 10.1093/brain/aww319, PMID: 28003242 PMC5837514

[ref11] Dubois B. Albert M. L. (2004). Amnestic MCI or prodromal Alzheimer’s disease? Lancet Neurol. 3, 246–248. doi: 10.1016/S1474-4422(04)00710-0 15039037

[ref12] Eavani H. Hsieh M. K. An Y. Erus G. Beason-Held L. Resnick S.. (2016). Capturing heterogeneous group differences using mixture-of-experts: application to a study of aging. NeuroImage 125, 498–514. doi: 10.1016/j.neuroimage.2015.10.045, PMID: 26525656 PMC5460911

[ref13] Edmonds E. C. Delano-Wood L. Clark L. R. Jak A. J. Nation D. A. McDonald C. R.. (2015). Susceptibility of the conventional criteria for mild cognitive impairment to false-positive diagnostic errors. Alzheimers Dement. 11, 415–424. doi: 10.1016/j.jalz.2014.03.005, PMID: 24857234 PMC4241187

[ref14] Edmonds E. C. Eppig J. Bondi M. W. Leyden K. M. Goodwin B. Delano-Wood L.. (2016). Heterogeneous cortical atrophy patterns in MCI not captured by conventional diagnostic criteria. Neurology 87, 2108–2116. doi: 10.1212/WNL.0000000000003326, PMID: 27760874 PMC5109943

[ref15] Fischl B. (2012). FreeSurfer. NeuroImage 62, 774–781. doi: 10.1016/j.neuroimage.2012.01.021, PMID: 22248573 PMC3685476

[ref16] Gibbons L. E. Carle A. C. Mackin R. S. Harvey D. Mukherjee S. Insel P.. (2012). A composite score for executive functioning, validated in Alzheimer’s Disease Neuroimaging Initiative (ADNI) participants with baseline mild cognitive impairment. Brain Imaging Behav. 6, 517–527. doi: 10.1007/s11682-012-9176-1, PMID: 22644789 PMC3684181

[ref17] Hu S. Yu W. Chen Z. Wang S. (2020). Medical image reconstruction using generative adversarial network for Alzheimer disease assessment with class-imbalance problem, in: *2020 IEEE 6th international conference on computer and communications (ICCC): IEEE*, 1323–1327.

[ref18] Jack C. R. Jr. Bernstein M. A. Borowski B. J. Gunter J. L. Fox N. C. Thompson P. M.. (2010). Update on the magnetic resonance imaging core of the Alzheimer’s disease neuroimaging initiative. Alzheimers Dement. 6, 212–220. doi: 10.1016/j.jalz.2010.03.004, PMID: 20451869 PMC2886577

[ref19] Jack C. R. Jr. Bernstein M. A. Fox N. C. Thompson P. Alexander G. Harvey D.. (2008). The Alzheimer’s disease neuroimaging initiative (ADNI): MRI methods. J. Magnetic Resonance Imag.: Official J. Int. Society for Magnetic Resonance Med. 27, 685–691. doi: 10.1002/jmri.21049, PMID: 18302232 PMC2544629

[ref20] Klein A. Tourville J. (2012). 101 labeled brain images and a consistent human cortical labeling protocol. Front. Neurosci. 6:33392. doi: 10.3389/fnins.2012.00171 PMC3514540 23227001

[ref21] Knopman D. S. Amieva H. Petersen R. C. Chételat G. Holtzman D. M. Hyman B. T.. (2021). Alzheimer disease. Nat. Rev. Dis. Prim. 7, 1–21. doi: 10.1038/s41572-021-00269-y 33986301 PMC8574196

[ref22] Kwak K. Giovanello K. S. Bozoki A. Styner M. Dayan E. (2021). Subtyping of mild cognitive impairment using a deep learning model based on brain atrophy patterns. Cell Reports Med. 2:100467. doi: 10.1016/j.xcrm.2021.100467, PMID: 35028609 PMC8714856

[ref23] Lo R. Y. Jagust W. J. (2012). Predicting missing biomarker data in a longitudinal study of Alzheimer disease. Neurology 78, 1376–1382. doi: 10.1212/WNL.0b013e318253d5b3, PMID: 22491869 PMC3345787

[ref24] Mueller S. G. Weiner M. W. Thal L. J. Petersen R. C. Jack C. Jagust W.. (2005). The Alzheimer’s disease neuroimaging initiative. Neuroimaging Clinics 15, 869–877. doi: 10.1016/j.nic.2005.09.008, PMID: 16443497 PMC2376747

[ref25] Murray M. E. Graff-Radford N. R. Ross O. A. Petersen R. C. Duara R. Dickson D. W. (2011). Neuropathologically defined subtypes of Alzheimer’s disease with distinct clinical characteristics: a retrospective study. Lancet Neurol. 10, 785–796. doi: 10.1016/S1474-4422(11)70156-9, PMID: 21802369 PMC3175379

[ref26] Nelson M. E. Jester D. J. Petkus A. J. Andel R. (2021). Cognitive reserve, Alzheimer’s neuropathology, and risk of dementia: a systematic review and meta-analysis. Neuropsychol. Rev. 31, 233–250. doi: 10.1007/s11065-021-09478-4, PMID: 33415533 PMC7790730

[ref27] Nettiksimmons J. DeCarli C. Landau S. Beckett L. Alzheimer’s Disease Neuroimaging Initiative (2014). Biological heterogeneity in ADNI amnestic mild cognitive impairment. Alzheimers Dement. 10, 511–521.e1. doi: 10.1016/j.jalz.2013.09.003, PMID: 24418061 PMC4092059

[ref28] Pan J. Lei B. Shen Y. Liu Y. Feng Z. Wang S. (2021). “Characterization multimodal connectivity of brain network by hypergraph GAN for Alzheimer’s disease analysis”, in: *Pattern recognition and computer vision: 4th Chinese conference, PRCV 2021, Beijing, China*, October 29–November 1, 2021, Proceedings, Part III 4: Springer, 467–478.

[ref29] Petersen R. C. Aisen P. S. Beckett L. A. Donohue M. C. Gamst A. C. Harvey D. J.. (2010). Alzheimer’s disease neuroimaging initiative (ADNI): clinical characterization. Neurology 74, 201–209. doi: 10.1212/WNL.0b013e3181cb3e25, PMID: 20042704 PMC2809036

[ref30] Petersen R. C. Morris J. C. (2005). Mild cognitive impairment as a clinical entity and treatment target. Arch. Neurol. 62:1160. doi: 10.1001/archneur.62.7.1160 16009779

[ref31] Shaw L. M. Vanderstichele H. Knapik-Czajka M. Clark C. M. Aisen P. S. Petersen R. C.. (2009). Cerebrospinal fluid biomarker signature in Alzheimer’s disease neuroimaging initiative subjects. Ann. Neurol. 65, 403–413. doi: 10.1002/ana.21610, PMID: 19296504 PMC2696350

[ref32] Shaw L. M. Vanderstichele H. Knapik-Czajka M. Figurski M. Coart E. Blennow K.. (2011). Qualification of the analytical and clinical performance of CSF biomarker analyses in ADNI. Acta Neuropathol. 121, 597–609. doi: 10.1007/s00401-011-0808-0, PMID: 21311900 PMC3175107

[ref33] Sun N. Mormino E. C. Chen J. Sabuncu M. R. Yeo B. T. the Alzheimer’s Disease Neuroimaging Initiative (2019). Multi-modal latent factor exploration of atrophy, cognitive and tau heterogeneity in Alzheimer’s disease. NeuroImage 201:116043. doi: 10.1016/j.neuroimage.2019.116043, PMID: 31344486

[ref34] Tanzi R. E. (2012). The genetics of Alzheimer disease. Cold Spring Harb. Perspect. Med. 2:a006296. doi: 10.1101/cshperspect.a006296 23028126 PMC3475404

[ref35] Ten Kate M. Dicks E. Visser P. J. Van Der Flier W. M. Teunissen C. E. Barkhof F.. (2018). Atrophy subtypes in prodromal Alzheimer’s disease are associated with cognitive decline. Brain 141, 3443–3456. doi: 10.1093/brain/awy264, PMID: 30351346 PMC6669409

[ref36] Van Oostveen W. M. de Lange E. C. (2021). Imaging techniques in Alzheimer’s disease: a review of applications in early diagnosis and longitudinal monitoring. Int. J. Mol. Sci. 22:2110. doi: 10.3390/ijms22042110, PMID: 33672696 PMC7924338

[ref37] Vivek S. Howard C. Lerch J. P. Evans A. C. Dorr A. E. Jehan K. N. (2006). Spatial patterns of cortical thinning in mild cognitive impairment and Alzheimer’s disease. Brain J. Neurol. 11:2885. 10.1093/brain/awl256 17008332

[ref38] Whitwell J. L. Petersen R. C. Negash S. Weigand S. D. Kantarci K. Ivnik R. J.. (2007). Patterns of atrophy differ among specific subtypes of mild cognitive impairment. Arch. Neurol. 64, 1130–1138. doi: 10.1001/archneur.64.8.1130, PMID: 17698703 PMC2735186

[ref39] Whitwell J. L. Shiung M. M. Przybelski S. A. Weigand S. D. Knopman D. S. Boeve B. F.. (2008). MRI patterns of atrophy associated with progression to AD in amnestic mild cognitive impairment. Neurology 70, 512–520. doi: 10.1212/01.wnl.0000280575.77437.a2, PMID: 17898323 PMC2734138

[ref40] Winblad B. Palmer K. Kivipelto M. Jelic V. Fratiglioni L. Wahlund L. O.. (2004). Mild cognitive impairment–beyond controversies, towards a consensus: report of the international working group on mild cognitive impairment. J. Intern. Med. 256, 240–246. doi: 10.1111/j.1365-2796.2004.01380.x 15324367

[ref41] Yang Z. Nasrallah I. M. Shou H. Wen J. Doshi J. Habes M.. (2021). A deep learning framework identifies dimensional representations of Alzheimer’s disease from brain structure. Nat. Commun. 12:7065. doi: 10.1038/s41467-021-26703-z, PMID: 34862382 PMC8642554

[ref42] Zhang B. Lin L. Wu S. (2021). A review of brain atrophy subtypes definition and analysis for Alzheimer’s disease heterogeneity studies. J. Alzheimers Dis. 80, 1339–1352. doi: 10.3233/JAD-201274, PMID: 33682711

[ref43] Zhao K. Zheng Q. Dyrba M. Rittman T. Li A. Che T.. (2022). Regional radiomics similarity networks reveal distinct subtypes and abnormality patterns in mild cognitive impairment. Advan. Sci. 9:e2104538. doi: 10.1002/advs.202104538, PMID: 35098696 PMC9036024

[ref44] Zuo Q. Lei B. Shen Y. Liu Y. Feng Z. Wang S. (2021). “Multimodal representations learning and adversarial hypergraph fusion for early Alzheimer’s disease prediction”, in: *Pattern recognition and computer vision: 4th Chinese conference, PRCV 2021, Beijing, China*, October 29–November 1, 2021, Proceedings, Part III 4: Springer, 479–490.

[ref45] Zuo Q. Wu H. Chen C. P. Lei B. Wang S. (2024). Prior-guided adversarial learning with hypergraph for predicting abnormal connections in Alzheimer’s disease. IEEE Transactions on Cybernetics., 1–14. doi: 10.1109/TCYB.2023.3344641 38236677

[ref46] Zuo Q. Zhong N. Pan Y. Wu H. Lei B. Wang S. (2023). Brain structure-function fusing representation learning using adversarial decomposed-VAE for analyzing MCI. IEEE Trans. Neural Syst. Rehabil. Eng. 31, 4017–4028. doi: 10.1109/TNSRE.2023.3323432 37815971

